# Effects of adult and egg predators on hatching plasticity of the pulmonate limpet

**DOI:** 10.1007/s00442-025-05712-5

**Published:** 2025-05-21

**Authors:** Yoko Wada, Keiji Iwasaki, Yoichi Yusa

**Affiliations:** 1 Faculty of Agriculture, Miyazaki University, Miyazaki, Japan; 2 Faculty of Letters, Nara University, Nara, Japan; 3 Faculty of Science, Nara Women’s University, Nara, Japan

**Keywords:** Hatching timing, Embryo, Egg predator, Adult predator, Intertidal rocky shore

## Abstract

In response to predation threats during the embryonic period, prey from diverse taxonomic groups exhibit plasticity in their hatching timing. In theory, predators of adult prey, as well as predators of eggs or embryos, can influence hatching timing. Similarly, not only embryos but also parents of prey can regulate hatching timing. However, research on the influence of adult predators and adult prey on hatching timing in species with separate predators for adults and eggs remains limited. To the best of our knowledge, no study has investigated this phenomenon in marine invertebrates under natural conditions. In this study, we investigated the effects of life-stage-specific predators (i.e., adult and egg predators) on the hatching timing of the pulmonate limpet (*Siphonaria sirius*), which undergoes planktonic development on an intertidal rocky shore. The presence of adult predators before and after egg-laying did not affect the hatching timing. Furthermore, while the egg predators present before egg-laying did not influence hatching timing, those present after egg-laying accelerated it. The results indicate that embryos, rather than their parents, determine hatching timing in response to their own predation risk. This finding highlights a strategy in which organisms with planktonic development rely on embryonic plasticity to mitigate strong predation risks during the egg stage. To understand how predation risk shapes predator–prey dynamics, it is critical to identify how predators, specific to each life-history stage of prey (such as adult and egg), interact with prey at different life-history stages during key events like reproduction.

**Supplementary Information:**

The online version contains supplementary material available at 10.1007/s00442-025-05712-5.

## Introduction

Given the significance of predation in interspecific interactions, prey species are expected to adapt to predation risks to increase their survival rates (Lima and Dill 1990; Sih and Moore 1993). Predation risks can induce dramatic shifts in prey feeding behavior (Schmitz et al. 1997; Wada et al. 2013; Matassa and Trussell 2014; Ho et al. 2019), reproductive behavior (Dulude-de Broin et al. 2020), habitat use (Laundré et al. 2010; Matassa and Trussell 2011; Kohl et al. 2018), morphology (Brönmark and Miner 1992; Kishida and Nishimura 2006), and physiology (Clinchy et al. 2013; Zanette and Clinchy 2019). These non-consumptive effects of predators, in addition to consumptive effects, influence the demography of prey species (Zanette et al. 2011). In particular, the embryonic period is one of the most vulnerable stages in the life history of prey (Fuiman and Magurran 1994). This vulnerability makes it likely that prey species evolve plasticity in their hatching timing in response to the risk of embryonic predation, as demonstrated in various taxonomic groups (Warkentin 2011a). In general, the presence of an egg predator (i.e., a predator that attacks eggs or embryos) tends to induce early hatching of embryos (e.g., amphibians: Warkentin 1999, 2000; Vonesh 2000; Chivers et al. 2001; Warkentin et al. 2001, fish: Kusch and Chivers 2004). However, a few studies have reported delayed hatching (e.g., crustaceans: Blaustein 1997; de Roeck et al. 2005; snails: Miner et al. 2010).

In theory, adult predators as well as egg predators can influence hatching timing. Similarly, hatching timing can be regulated not only by embryos but also by their parents. However, to the best of our knowledge, how adult predators and adult prey influence hatching timing in species with distinct predators for adult and egg prey remains unexplored. While egg predators have a direct impact on embryo hatching control (Warkentin 2011a), the adult predator may indirectly affect hatching timing through changes in parental investment in eggs. Even in species without post-ovipositional parental care (Li 2002), parents detecting their predator cues can affect embryo hatching plasticity through various pathways. For example, parents detecting their own predator cues change their foraging behavior (Downes 2001; Wada et al. 2013), leading to poorer physical conditions (Ball and Baker 1996; Abrams and Rowe 1996) that affect their egg and offspring performance. In a fish species, adults in poor body conditions tend to lay fewer and smaller eggs (Bennett and Murray 2014), whereas the nutritional condition of adults is linked to the egg size in a marine snail (Krug 2001). Moreover, animals, particularly iteroparous species, often adjust their reproductive timing to optimize their own fitness, sometimes at the cost of their offspring (Warner 1998). As a result, predation pressure on adults can influence egg quality through incomplete mating or egg-laying, ultimately leading to reduced hatching success (Guo et al. 2017). On the other hand, when parental and offspring environments are complementary, parents can anticipate the conditions their offspring will encounter, making the transmission of parental risk experience to offspring adaptive (Bell and Hellmann 2019). Parents exposed to predation stress increase egg size with higher cortisol content to provide larvae with an advantage, as observed in three-spined sticklebacks (Giesing et al. 2011) and mouthbrooding cichlids (Segers and Taborsky 2012).

In contrast to terrestrial and freshwater species, the effects of predation risk on hatching timing in marine invertebrates have been poorly investigated (Collin et al. 2016). Existing studies have primarily focused on species with direct development that lack a planktonic larval phase (Miner et al. 2010; Donelan and Trussell 2015, 2018a, b). In direct-developing species, eggs and offspring experience similar environmental conditions to those of their parents. Consequently, parents can sufficiently predict the environments their eggs and offspring will encounter. In this case, the environments of parents could influence the hatching timing and phenotype of their offspring (i.e., parental effects: Burgess and Marshall 2014). Notably, in a direct-developing marine snail, parents exposed to a predator cue were shown to influence the earlier hatching of their offspring (Donelan and Trussell 2018a). In contrast, species with planktonic larvae experience distinctly different environments between parental (benthos) and larval (neuston) phases, making parental effects less likely. The adjustment of hatching timing has received little attention in marine invertebrates with planktonic larvae, except studies on marine gastropods, where physical damage to egg masses simulating predator risk induces rapid hatching (Strathmann et al. 2010), and on mud snails, where cues from egg predators alter egg capsule morphology without affecting larval hatching timing (Schwab and Allen 2014). Although adult mud snails have been observed to modify the deposition sites of egg capsules at higher locations in the presence of benthic egg predators (Harmon and Allen 2018), their impact on hatching timing has not been investigated. Given that many invertebrates develop via planktonic larval stages after hatching (Oyarzun and Strathmann 2011), it is important to investigate the impacts of predators on hatching timing and the potential influence of parental effects. Additionally, understanding how embryos detect predation risk and adjust their hatching timing merits further investigation in species with planktonic larvae.

Given the risk posed by egg predators, early hatching results in smaller, less developed larvae, which are more vulnerable to larval predators (Warkentin 1995, 1999; Ituarte et al. 2014; Willink et al. 2014; Delia et al. 2019). Therefore, there may be a trade-off between the vulnerability to the egg predator and vulnerability to the larval predator, highlighting the importance of monitoring hatching timing in natural environments in the presence of larval predators. Furthermore, since intertidal organisms are exposed to fluctuating and stressful abiotic environmental conditions (Przeslawski 2004; Collin et al. 2016), such as temperature (Rodriguez et al. 1991), desiccation (Przeslawski 2004), salinity (Armstrong et al. 2013), oxygen availability (Fernandes and Podolsky 2011), and ultraviolet radiations (Gallardo et al. 2013), abiotic conditions play a critical role in determining hatching plasticity (Warkentin 2011b). These abiotic factors can interact with predation risk, influencing the optimal hatching timing (Touchon et al. 2011). However, the effects of predators, particularly in aquatic ecosystems, have primarily been investigated in laboratory settings with minimal fluctuation in abiotic conditions.

This study investigated the hatching plasticity of the marine pulmonate limpet, *Siphonaria sirius*, in the presence of their adult and egg predators on an intertidal rocky shore. *Siphonaria sirius* is hermaphroditic (Hodgson 1999) and an iteroparous species (Iwasaki 1995), mating early in the morning during neap tides in summer, and laying spiral-shaped egg masses on rock surfaces (Electronic Supplemental Material, EMS Fig. S1(c)) one day after mating (Iwasaki 1995; Wada and Yusa 2021). Each egg mass contains approximately 2600 egg capsules, each containing an egg (Wada and Yusa 2021). The eggs develop into an advanced veliger stage within the egg mass before hatching as planktonic larvae. Thus, in the presence of egg predators, the embryos may have the option to hatch early, soon after reaching an early veliger stage, or to wait until they develop into fully developed veligers. The duration of the planktonic phase is unknown for *S. sirius*, but that of the congener *S. denticulata* is estimated to be approximately 10 weeks (Creese 1980). The carnivorous snail *Reishia clavigera* (“C type” sensu Abe 1985) is a major predator of adult *S. sirius*, particularly during summer, the reproductive season of *S. sirius* (Wada et al. 2017). *Reishia clavigera* has not been observed attacking the egg masses of *S. sirius*, but it has been found to feed on neritid eggs packed in hard capsules (Fukumori et al. 2013). Our previous observations indicated that the polyphagous snails *Tenguella musiva* and *Drupella margariticola* were the primary egg predators of *S. sirius* at the study site (Wada and Yusa 2021). *T. musiva* mainly feeds on sessile invertebrates such as barnacles and bivalves (Abe 1989), whereas *D. margariticola* is known to prey on caenogastropod eggs (Abe 1983).

Given the lack of information on predation by these snails, we investigated the predation pressure exerted by the *R. clavigera*, *T. musiva*, and *D. margariticola* on egg masses of *S. sirius* in a preliminary experiment (ESM S1). The result showed that *R. clavigera* preys only on adult *S. sirius,* not their egg masses. Building on this, we conducted two factorial field experiments to examine the responses of both adults and embryos of *S. sirius* to adult and egg predators. The first experiment assessed the effects of predation risks from adult and egg predators on the hatching timing of *S. sirius* embryos during the reproductive event (from just before mating to the hatching of eggs). The second experiment aimed to investigate when and who (*S. sirius* adult or embryo) alter the hatching timing by providing egg predation risk before and/or after egg-laying of *S. sirius*. We hypothesized that both adults and embryos of *S. sirius* would respond to their own predators, rather than to the predators of other life stages. Specifically, we predicted that adults would adjust the embryo hatching timing by reducing their investment in each egg due to energy constraints in the presence of adult predators (Wada et al. 2017). In contrast, embryos were expected to detect their own predation risk and hatch earlier in response to egg predation risk after egg-laying.

## Materials and methods

### Experimental setup

We performed two field experiments in the intertidal area near Seto Marine Biological Laboratory, Shirahama, Wakayama, Japan (33°41 N, 135°20 E) to achieve the following objectives: (1) to investigate whether cues from adult and egg predators of *S. sirius* during the reproductive period affects the embryo hatching timing, and (2) to identify whether parents or embryos of *S. sirius* control the hatching timing. Six experimental blocks in Experiment 1 and eight blocks in Experiment 2 were established, based on a random block design (EMS Fig. S1(a)). These blocks varied slightly in tidal levels and wave exposure. The mean [± SD] distances between blocks were 3.97 [4.34] m in Experiment 1 and 3.14 [2.25] m in Experiment 2. Each block contained five experimental plots represented by individual sandstone rocks (EMS Fig. S1(b)) spaced at least 1 m apart. A total of 30 rocks were used in Experiment 1 and 40 rocks in Experiment 2.

To mitigate the effects of intraspecific competition, rocks with excessively high densities of adult *S. sirius* were excluded from selection as experimental plots. Five treatment groups were assigned to the five experimental plots in each block for both experiments. No significant differences were found in the number of adult *S. sirius* in the experimental plots and plot size among the treatment groups (Experiment 1: mean [± SD] number of individuals = 44.17 [28.72] (likelihood χ^2^_4_ = 0.15, *P* = 0.997), plot area = 0.45 [0.14] m^2^ (likelihood χ^2^_4_ = 2.66, *P* = 0.62), Experiment 2: number of individuals = 45.27 [22.17] (likelihood χ^2^_4_ = 4.13, *P* = 0.39), plot area = 0.32 [0.12] m^2^ (likelihood χ^2^_4_ = 4.85, *P* = 0.30; generalized linear mixed models (GLMM) with Poisson and gamma distributions (ln-link function) for the number of individuals and plot area, respectively, block included as a random factor).

To minimize the influence of other mobile animals on the reproductive events and hatching timing of *S. sirius*, all mobile animals except for *S. sirius* were removed from experimental plots before starting the experiments. A 5-cm-wide copper-infused paint barrier (Denka, Tokyo, Japan) was applied along the plot edges (Wada et al. 2013) to prevent re-entry. We measured the shell length of *S. sirius* (5–15 individuals per plot in Experiment 1 and 1–7 individuals per plot in Experiment 2) and found no significant differences among the five treatment groups (Experiment 1: [mean ± SD] = 14.85 ± 3.76 mm, likelihood χ^2^_4_ = 1.20, *P* = 0.88; Experiment 2: 14.64 ± 3.22 mm, likelihood χ^2^_4_ = 1.41, *P* = 0.84; GLM analyses using a gamma distribution (ln-link function)).

We attached one small mesh cage (25 mm tall, 77 mm in diameter, ESM Fig. S1(b)) to all plots except for Natural plots (see below) using glue (Konishi Corporation, Osaka, Japan). These cages contained the predators and experimental victims (adult individuals of *S. sirius* and/or egg masses of *S. sirius*) to simulate predation risk cues. The response of adult individuals of *S. sirius* and/or embryos of *S. sirius* outside the cage was then evaluated.

### Experiment 1

In Experiment 1, we assessed the effects of adult (*R. clavigera*) and egg (*T. musiva*) predators on the hatching timing of *S. sirius* eggs during the reproductive period (10–17-Sep-2014: just before mating until hatching). *Tenguella musiva* was selected as the egg predator due to its higher abundance in the field (ESM S1). Preliminary experiments revealed that prolonged exposure of adult *S. sirius* to the adult predator cues had no significant effect on the hatching timing of *S. sirius* eggs (ESM S2). Similarly, the duration (either > 1 month or 4 days) of adult *S. sirius* exposure to the egg predators did not affect the hatching timing of *S. sirius* eggs (ESM S3). Based on these results, this study focused on short-term predator treatments. In this experiment, the egg masses were observed on 12-Sep-2014 across all experimental plots, with most (85.7%, average [LSE, USE] per plot: 6.10 [5.03, 7.40]) egg masses laid during the morning low tide of 12-Sep-2014. A total of 7.12 [5.92, 8.57] egg masses were laid throughout the reproductive period. To ensure uniform environmental conditions, only egg masses laid on 12-Sep were used for the experiments. Those laid later were included only in the total egg mass counts.

We used a fully crossed design with two factors: adult predator (*R. clavigera*) statuses (absence or presence) and egg predator (*T. musiva*) statuses (absence or presence), resulting in four treatment groups: Adult predator only, Egg predator only, both (Adult predator × Egg predator), and without adult predator and egg predator (Control). To estimate the natural predation rates on *S. sirius* adult and egg masses, one “Natural” plot per block was established without paint barriers or cages. Each block included five plots, with a total of six blocks used in the experiment. For Adult predator treatment, six adult predators were placed in each cage, based on the mean number of adult predators observed in Natural plots ([mean ± SD]: 5.83 ± 0.75 individuals) before this experiment (between 28-Aug and 05-Sep-2014). To ensure that predation risk cues were emitted by adult predators, several adult *S. sirius* were placed in the cages as prospective prey items (referred to as experimental victims). The number of experimental victims (adult *S. sirius*) was standardized based on the consumption rate of adult *S. sirius* (13.85% of total adult individuals in each plot). This consumption rate was estimated as the average mortality of adult *S. sirius* in Natural plots (where adult predators could access *S. sirius*) minus that in Control plots (where adult predators were excluded) during the previous mating–hatching period (28-Aug–05-Sep-2014). As a result, the number of experimental victims (adult *S. sirius*) placed in each cage ranged from two to 13, with a mean [± SD] of 5.98 [5.19]. The treatment began on 10-Sep-2014, 2 days before most egg masses had been laid in each experimental plot.

For Egg predator treatment, four or five egg masses of *S. sirius* (mean [± SD]: 4.34 [0.76]) were introduced into each cage as experimental victims, depending on the total number of egg masses laid in each plot during the previous egg-laying–hatching period (29-Aug–05-Sep-2014). The exact number in each plot was determined by applying a consumption rate of 44.17% to the total egg masses laid in each plot during the same period. This consumption rate was calculated as the proportion of egg masses consumed by egg predators to the total egg masses laid in Natural plots during the same period. The egg masses were pressed gently against the substrate to adhere. Then, six egg predators were introduced in the same cage, based on the average number of egg predators (*T. musiva* and *D. margariticola*) observed in Natural plots between 28-Aug and 05-Sep-2014 ([mean ± SD]: 5.67 ± 0.82 individuals). The experiment began immediately after collecting the egg masses from rocks at higher tidal levels than the experimental site on 10-Sep-2014, 2 days before the peak of egg masses laying at the experimental plots.

Previous studies on the combined effects of predators (i.e., Adult predator × Egg predator treatments in the present study) have confounded predator composition with total predator density (Relyea 2003). To address this, we conducted a preliminary experiment comparing the effects of additive and halved predator treatments on the hatching timing of *S. sirius*. The results showed no significant differences in the hatching timing between the two treatments (ESM S4). Based on these results and cage space limitations, we adopted the halved treatment for Adult predator × Egg predator treatments, using three adult predators and three egg predators per cage. The experimental victims consisted of 6.92% of the total adult *S. sirius* in each experimental plot (ranging from 1 to 6 individuals, mean ± SD: 3.30 ± 2.06) and 22.09% of the total egg masses laid in each experimental plot before the reproductive period (ranging from 1 to 2 egg masses, mean ± SD: 1.97 ± 0.58). We confirmed that *R. clavigera* and *T. musiva* were consuming adult *S. sirius* and egg masses, respectively.

We observed the presence or absence of egg masses once a day during daytime low tide until 12-Sep. One egg mass per experimental plot was collected (i.e., *n* = 6 egg masses per treatment group) on 12-Sep and preserved in 10% seawater formalin to determine the egg number and size (ESM S5). We monitored larval development and hatching timing twice daily during low tide from 12-Sep to 17-Sep. Egg masses were numbered on 12-Sep, and one marked egg mass per plot was collected during each survey for microscopic examination of developmental stages. We avoided continuous observations of the same egg mass because cutting a portion of an egg mass for examination might be perceived as a predation cue for remaining embryos. Instead, different egg masses were collected intact for developmental monitoring, providing up to six replicates per treatment group across six blocks. Hatching was confirmed when only egg capsules remained in the egg masses, and all marked egg masses hatched by 17-Sep-2014. Seawater temperature was recorded during each survey to monitor environmental conditions.

### Experiment 2

In the second experiment (29-Aug–06-Sep-2015), we investigated whether adult *S. sirius* or their embryos regulate hatching timing in response to the egg predator (*T. musiva*). The egg masses were observed on 2-Sep-2015 across all experimental plots. The mean [LSE, USE] numbers of egg masses laid per plot on the first day (during the morning neap tide on 2-Sep-2015) and the following day (during the morning neap tide on 3-Sep-2015) were 6.82 [6.37, 7.30] and 8.06 [7.58, 8.58], respectively, indicating that most egg masses were laid on 2-Sep. To ensure consistent conditions, only the egg masses laid on 2-Sep were used in the experiment, while those laid later were included solely in the egg mass counts.

To expose both adults and embryos living outside the cages to egg predator cues, we placed egg predators and experimental victims (egg masses) inside the cages in the plots at two distinct time points: before (Before egg-laying treatment) and after (After egg-laying treatment) the egg-laying peak. In Before egg-laying treatment, the egg predators and egg masses were introduced in the cage on 29-Aug-2015, prior to the egg-laying of *S. sirius* individuals, and removed before the peak of egg-laying (2-Sep-2015). In After egg-laying treatment, the egg predators and egg masses were introduced only after egg masses were laid on 2-Sep-2015.

We used a fully crossed design with these two treatments, resulting in four treatment groups: Before egg-laying only, After egg-laying only, both (Before × After egg-laying), and neither treatment (Control). For Before × After egg-laying treatments, the predators and experimental victims were introduced before egg-laying and remained in the cages until all marked egg masses had hatched (06-Sep-2015). These treatment groups were replicated across eight blocks, and one Natural plot was included in each block as in Experiment 1.

The number of egg predators in the cages was determined based on the mean number observed in eight Natural plots (mean [± SD]: 4.63 [0.74]) before this experiment (17–22-Aug-2015). The number of experimental victims (egg masses) in each cage was calculated by multiplying the total number of egg masses laid in each plot during the previous egg-laying–hatching period (17–22-Aug-2015) by the consumption rate of egg masses (47.91%), estimated as the proportion of preyed egg masses to the total laid in Natural plots during the same period. The mean [± SD] number of egg masses per cage was 4.85 [4.30] for Before egg-laying treatment (range 1–13), 4.49 [2.43] for After egg-laying treatment (range 2–9), and 4.13 [2.84] for Before × After egg-laying treatment (range 1–9). The egg masses were collected from higher tidal areas, where egg-laying occurred before the experimental plots.

Similar to Experiment 1, we observed the developmental stages and hatching timing of marked egg masses at each low tide (twice a day). Additionally, the egg masses collected during the first survey after egg-laying (on 2-Sep-2015) were used to measure the weight of egg masses and estimate egg number and size (ESM S5). Seawater temperature was measured at each low tide from egg-laying (2-Sep) to hatching (6-Sep).

If the presence of egg predators before egg-laying affects the hatching timing, adults should control the hatching timing, because the embryos have not yet been formed, and thus any response must originate from the adults. If the presence of the egg predators after egg-laying influences the hatching timing, then the embryos should control their own hatching timing, as the adults do not exhibit parental care and hence cannot exert control over hatching timing after eggs are laid.

### Data and statistical analyses

All analyses were performed using R software (version 4.1.1; R Development Core Team 2021). Generalized linear mixed models (GLMMs) were used to analyze the effects of the presence of (1) adult and egg predators (for Experiment 1) and (2) egg predators before and after egg-laying (Experiment 2) on the number of egg masses laid on the first day (12-Sep-2014 in Experiment 1, 2-Sep-2015 in Experiment 2) in each plot, total number of egg masses in each plot, weight of whole egg mass, egg number per mg, egg size, and hatching timing of *S. sirius*. The models included treatments (i.e., Adult and Egg predator in Experiment 1 and Before and After egg-laying in Experiment 2) and their interactions as fixed factors. Block and rock identities were included as random factors for egg mass number, egg mass weight, egg number, and hatching timing, while block and rock and egg mass fragment identities were included as random factors for egg size. The ln(weight of each egg mass fragment) was included as an offset term for analyses of egg number. The number of egg masses and eggs, and the egg mass weight and egg size were analyzed using a Poisson and gamma distributions (ln-link function), respectively. Hatching timing was defined as the number of low tides (i.e., emergence from seawater) experienced by each egg mass before hatching. This was because data on embryonic development and hatching could only be collected during low tides. The number of low tides was analyzed using a Poisson distribution (ln-link function). Hatching data for egg masses that completely disappeared during our visits to the study site at low tides were excluded, as it was unclear whether they hatched or were dislodged by wave actions. Consequently, data on developmental stage and hatching timing were unavailable for 4 out of 24 experimental plots in Experiment 1 (Adult predator only in block no. 2, and Egg predator only, Adult and Egg predator, and Control in block no. 6) and 4 out of 32 experimental plots in Experiment 2 (Before egg-laying only and After egg-laying only in block no. 5, and After egg-laying only and Before and After egg-laying in block no. 8). The final number of egg masses investigated for hatching timing was 31 in Experiment 1 (Adult predator: *n* = 7, Egg predator: *n* = 7, Adult × Egg predator:* n* = 8, Control: *n* = 9) and 67 in Experiment 2 (Before egg-laying: *n* = 16, After egg-laying: *n* = 18, Before × After egg-laying: *n* = 18, Control: *n* = 15). The significance of the main effects and interactions was determined using likelihood *X*^2^ tests. To facilitate interpretation, we adjusted the results for specific factors by accounting for the effects of the other components of the statistical models using least-squares mean values (Milliken and Johnson 2009).

## Results

### Experiment 1

The average [± SD] seawater temperature was 27.73 [1.10] °C. Neither Adult predator nor Egg predator treatments affected the number of egg masses (Table 1). The weight of the egg masses did not differ between treatments (20.06 [17.94, 22.43] mg, Table 1). After accounting for the effect of the weight of each egg mass fragment as an offset term, the number of eggs was unaffected by treatments (Table 1, overall mean [LSE, USE] = 94.22 [91.46, 97.05]). Multiplying this by the weight of the whole egg mass, the number of eggs per egg mass was estimated to be 1,890. The size of eggs also did not differ between treatments (Table 1, overall mean = 6.13 × 10^5^ [5.81 × 10^5^, 6.47 × 10^5^] µm^3^).

**Table 1:** Statistics for effects of adult (presence vs. absence) and egg predators (presence vs. absence) on the number of egg masses laid on the first day (12-Sep-2014) in each plot and total number of egg masses in each plot, weight of whole egg mass, egg number per fragment, egg size, and the hatching timing of *Siphonaria sirius* in Experiment 1

Factors	Egg mass number		Egg mass weight	Egg number	Egg size	Hatching timing
	Day 1	Total				
Adult predator	*χ*^2^_1_ = 1.23	*χ*^2^_1_ = 2.42	*χ*^2^_1_ = 3.16	*χ*^2^_1_ = 1.26	*χ*^2^_1_ = 1.04	*χ*^2^_1_ = 0.0005
Egg predator	*χ*^2^_1_ = 2.45	*χ*^2^_1_ = 0.68	*χ*^2^_1_ = 2.50	*χ*^2^_1_ = 0.22	*χ*^2^_1_ = 1.58	***χ***^**2**^_**1**_ = 6.71*
Adult × Egg predator	*χ*^2^_1_ = 0.48	*χ*^2^_1_ = 0.03	*χ*^2^_1_ = 0.94	*χ*^2^_1_ = 1.64	*χ*^2^_1_ = 2.23	*χ*^2^_1_ = 0.94

Bolded score with an asterisk (*) indicates a significant effect (*P* < 0.01)

**Fig. 1:**
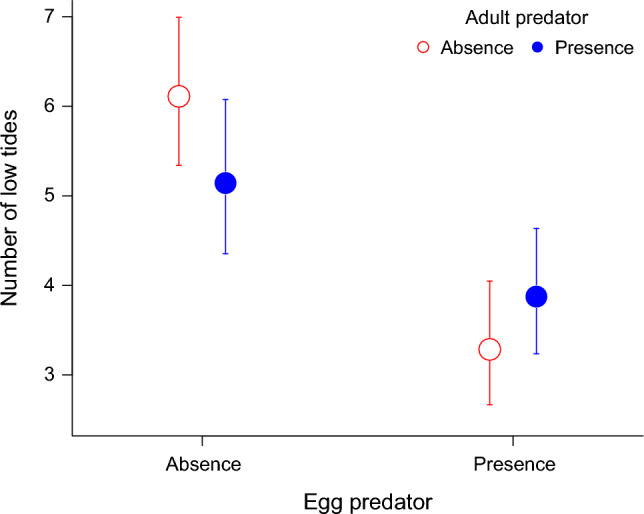
The number of low tides before hatching (i.e., hatching timing) of *Siphonaria sirius* embryos (mean ± SE) in the presence (filled symbols) or absence (open symbols) of the adult predator *Reishia clavigera* and the presence (right side) or absence (left side) of the egg predator *Tenguella musiva.* (sample sizes: presence of only adult predator, *n* = 7; only egg predator, *n* = 7; both adult and egg predator,* n* = 8; neither predator (Control), *n* = 9)

Hatching timing, measured as the number of low tides, was significantly shorter with Egg predator treatment (mean [LSE, USE] = 3.56 [3.10, 4.09]) compared to the absence of Egg predator treatment (mean [LSE, USE] = 5.58 [5.02, 6.22]). However, there was no significant difference between treatments with or without adult predators (Fig. 1, Table 1). Thus, the larvae hatched 2.02 low tides (1.01 days) earlier in the presence of egg predators than in their absence.

Additionally, we observed the following developmental stages in the eggs during the low tides: transition from blastula to gastrula at the first low tide (0.5 d after egg-laying), from gastrula to trochophore at the second low tide (1 d after egg-laying), the early veliger stage at the third low tide (1.5 d after egg-laying), and the advanced veliger stage at the fourth low tide (2 days after egg-laying). At 0.5 d after egg-laying, all egg masses under Adult predator × Egg predator treatment contained embryos at the gastrula stage. However, in other treatments, some egg masses contained embryos still at the blastula stage (Fig. S3), with no significant difference in developmental stage among treatments (GLM: *X*^2^_2_ = 0.66, *P* = 0.72). At 1 d after egg-laying, all egg masses observed contained trochophore-stage embryos, except those in Control. Thus, although there was a tendency for slightly faster development under Adult predator × Egg predator treatment, no clear differences in developmental speed were observed.

### Experiment 2

**Fig. 2:**
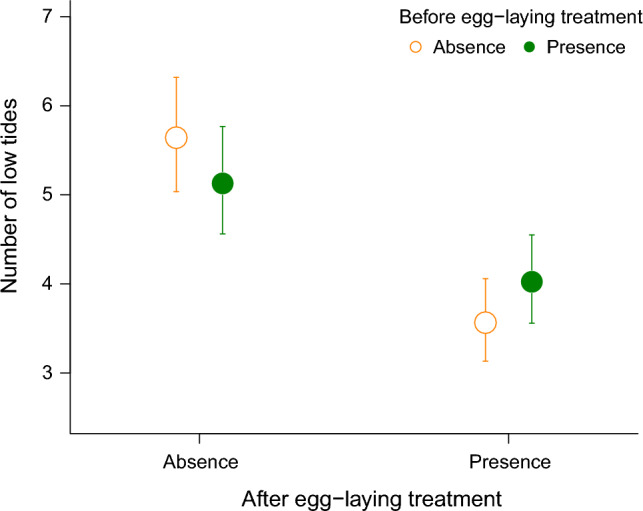
The number of low tides before hatching (i.e., hatching timing) of *Siphonaria sirius* embryos (mean ± SE) in the presence (filled symbols) or absence (open symbols) of the egg predator *Tenguella musiva* before egg-laying and the presence (right side) or absence (left side) of egg predator after egg-laying (sample sizes: presence of only before egg-laying, *n* = 16; only after egg-laying, *n* = 18; both before and after egg-laying, *n* = 18; neither (Control), *n* = 15)

The average [± SD] seawater temperature was 26.57 [1.39] °C. Neither Before egg-laying nor After egg-laying treatment affected the number of egg mass, egg mass weight, egg number, and egg size; however, hatching timing was reduced under After egg-laying treatment (Table 2). The weight of the egg mass and the number of eggs per unit weight were 22.94 [20.29, 22.94] mg and 86.29 [80.70, 92.26], respectively. Thus, the total number of eggs in the egg mass was estimated to be 1,979. The mean egg size was 6.17 × 10^5^ [6.16 × 10^5^, 6.18 × 10^5^] µm^3^. Hatching occurred 1.58 low tides (almost 0.79 days) earlier in the presence of egg predators after egg-laying compared to their absence, whereas it did not depend on the presence of egg predators before egg-laying (Fig. 2, Table 2).

**Table 2:** Statistics for effects of egg predators on the number of egg masses laid on the first day (2-Sep-2015) in each plot and total number of egg masses in each plot, weight of whole egg mass, egg number per fragment, egg size, and the hatching timing of *Siphonaria sirius* before and after egg-laying in Experiment 2

Factors	Egg mass number		Egg mass weight	Egg number	Egg size	Hatching timing
	Day 1	Total				
Before	*χ*^2^_1_ = 2.63	*χ*^2^_1_ = 1.53	*χ*^2^_1_ = 0.16	*χ*^2^_1_ = 0.003	*χ*^2^_1_ = 1.37	*χ*^2^_1_ = 0.01
After	*χ*^2^_1_ = 0.002	*χ*^2^_1_ = 0.02	*χ*^2^_1_ = 0.64	*χ*^2^_1_ = 1.42	*χ*^2^_1_ = 1.39	***χ***^**2**^_**1**_ = 8.89*
Before × After	*χ*^2^_1_ = 0.17	*χ*^2^_1_ = 0.98	*χ*^2^_1_ = 0.63	*χ*^2^_1_ = 2.94	*χ*^2^_1_ = 0.78	*χ*^2^_1_ = 0.85

“Before” and “After” indicate the treatments that the presence of the egg predator and the egg mass before and after egg-laying, respectively. Bolded score with an asterisk (*) indicates a significant effect (*P* < 0.01)

The developmental stages from egg-laying to hatching (Fig. S4) were observed as follows: at 0.5 days after egg-laying, all embryos remained at the blastula stage under Before egg-laying × After egg-laying treatments. Only one egg mass under other conditions contained embryos that had reached the gastrula stage, while embryos in other egg masses remained at the blastula stage. At 1 day after egg-laying, the number of egg masses containing embryos at the gastrula stage did not differ among the treatments (GLM; Before: *X*^2^_1_ = 1.07, *P* > 0.3, After: *X*^2^_1_ = 0.60, *P* > 0.4, Before × After: *X*^2^_1_ = 0.12, *P* > 0.7). Thus, there were no significant differences in developmental speed under different predation conditions.

## Discussion

We examined the effects of adult predators (*R. clavigera*) and egg predators (*T. musiva*) on the hatching timing of *S. sirius* during the reproductive period (just before mating to hatching) in Experiment 1. As a result, only the egg predators accelerated the hatching of the embryos by approximately 1 day (Fig. 1). In this study, the presence of any predators did not affect the number or size of eggs, nor the number of egg masses obtained from all experimental plots. Furthermore, to determine whether the parents or the embryos are responsible for the early hatching caused by the egg predators, a cue from the feeding egg predators was provided before and after egg-laying (Experiment 2). The results demonstrated that the cues from the feeding egg predators after egg-laying accelerated the hatching of the embryos by approximately 1 day (Fig. 2). This indicates that the embryos themselves responded to the cues from the feeding egg predators to determine their hatching timing. Approximately 50% of the egg predators in the field prey on *S. sirius* egg masses (EMS S1), and nearly 50% of the egg masses are consumed by egg predators during the embryonic period. Additionally, these predators can consume approximately 70% of an egg mass within half a day (ESM S1). Therefore, it is advantageous for *S. sirius* embryos to respond to the cues from feeding egg predators and hatch earlier. Several species with direct development can detect predators during their relatively long embryonic stage and modify their morphology and behavior (Dalesman et al. 2015; Solas et al. 2015). In the barnacle *Balanus glandula*, which has a planktonic larval stage, the embryos hatch when a predator crab consumes the brooding adults and breaks the barnacles’ test (Branscomb et al. 2014). Similarly, in some nudibranch gastropods with the planktonic larval stage, hatching is accelerated in response to the mechanical disturbance by a portunid crab (Strathmann et al. 2010). Notably, the present study is the first to demonstrate that embryos themselves detect predation risks through chemical cues and alter their hatching timing in a marine invertebrate with a planktonic larval stage.

We hypothesized that the presence of the adult predators might affect the hatching timing of *S. sirius* embryos due to changes in the egg size and quality. However, unlike those observed fish (Giesing et al. 2011) and freshwater snails (Guo et al. 2017), we observed that adult predators did not affect egg size, egg number, or hatching timing. *Reishia clavigera* is a major predator of adult *S. sirius,* particularly during the summer reproductive period. A reduction in feeding and growth rate, as well as an increase in mortality, was observed under predation pressure during the reproductive season (Wada et al. 2013, 2017). These results suggest that higher mortality during the reproductive season may be due to the lack of changes in their energy investment in reproduction, despite decreases in feeding and growth rate in response to predator avoidance.

In species that undergo direct development, where parents and offspring are exposed to common species, the experiences that parents have with their predators may influence the size and behavior of the offspring (Donelan and Trussell 2015, 2018a, b; des Roches et al. 2022). In contrast, for animals with a planktonic larval stage, such as *S. sirius*, parental experiences with their own predators are unlikely to affect the hatching timing of their offspring if the predators of the adult *S. sirius* do not feed on their egg masses. Moreover, it has been reported that parents have the potential to affect offspring survival when parents’ environment is a good predictor of offspring environment (Burgess and Marshall 2014). For rocky shore animals with a planktonic larval stage, the benthic environments of adults and the planktonic environment of offspring are quite different. Notably, the duration of the planktonic phase in *S. sirius* is estimated to be several months, meaning that the offspring disperse long distances. The temporal and spatial unpredictability of offspring environments is unlikely to result in the evolution of parental effects.

Additionally, we considered the possibility that the presence of egg predators could affect the timing of embryo hatching by influencing the timing of parental egg-laying, as reported in fish (Šmejkal et al. 2018). The advantage of shifting the egg-laying timing would occur if three conditions were met: (1) parents can detect the presence of egg predators; (2) egg predators have definite activity times; (3) eggs are already dispersed or hidden in the environment when egg predators resume their feeding activity. For *S. sirius*, even if the adult individuals could detect the egg predators, they might not be able to avoid egg predation by altering their spawning timing, since the predators exhibited high predation rates throughout the day and the embryos required at least 2 days to develop before hatching (Wada and Yusa 2021). As described above, our experiments confirmed the absence of parental effects under egg predation pressure during a single reproductive period (from mating to hatching). However, we cannot entirely rule out the possibility that parental effects, such as increased mating or spawning behavior, could emerge in subsequent reproductive periods.

Compared to closely related species, the embryonic period of *S. sirius* is notably short (Wada and Yusa 2021). This study suggests that egg predators may trigger earlier hatching in *S. Sirius,* highlighting the potential evolution of hatching timing plasticity in response to strong predation pressure on eggs. Additionally, it was confirmed that embryonic development progressed at a similar speed under all the treatments, although the exact developmental stage at hatching could not be ascertained. Therefore, the embryos did not accelerate their development in response to egg predators, and the earliest hatchlings emerged as early veligers capable of swimming. However, the optimal hatching timing is affected not only by predation risk during the embryonic period but also by risk during the planktonic larval period. Several studies on marine organisms with a planktonic larval stage have shown that mortality is higher during the larval stage than during the embryonic period (Morgan 2020; Strathmann 1985, 2007). Because early hatching can affect the vulnerability to predators immediately after hatching when mobility is low (Warkentin 1995), the vulnerability to larval predators must be examined. Moreover, early hatching affects the duration of the planktonic phase when released at or near metamorphic competence (Oyarzun and Strathmann 2011). However, the larval period is expected to last for several months in *S. sirius*, as the embryos hatch in summer (Iwasaki 1995; Wada and Yusa 2021) and settlement occurs in autumn (Wada et al. 2017). Thus, earlier hatching of one day will have little effect on the duration of the planktonic phase in *S. sirius*. Such a trade-off between predation risks during the embryonic and larval periods should be further explored.

In this study, we examined the effects of adult and egg predators on the timing of embryo hatching in a species with planktonic development, a common developmental mode among many marine invertebrates. Our findings reveal that egg predators influence hatching timing, with embryos themselves determining the timing, independent of parental influence. When adults, eggs, and larvae do not share a common predator, interactions between each life-history stage of prey and its specific predator can influence reproductive events in different ways. Understanding how stage-specific predators (e.g., those targeting adults and eggs) influence hatching timing and reproductive strategies is essential for elucidating predator–prey dynamics across life-history stages.

## Supplementary Information

Below is the link to the electronic supplementary material.

Supplementary file1 (DOCX 1302 KB)

## Data Availability

Data available from the Dryad Digital Repository: 10.5061/dryad.0000000dj.
