# Supplementary material for: Effects of adult and egg predators on hatching plasticity of the pulmonate limpet
Source: Oecologia. 2025 May 21;207(6):86. doi: 10.1007/s00442-025-05712-5 (PMC12095334; doi:10.1007/s00442-025-05712-5)
Supplement: Supplementary file 1 — Supplementary file1 (DOCX 1302 KB) [file 442_2025_5712_MOESM1_ESM.docx]

**Electronic Supplemental Material**

**Effects of adult and egg predators on hatching plasticity of the pulmonate limpet**

Yoko Wada*, Keiji Iwasaki, Yoichi Yusa

* Corresponding Author: yoko_wada@miyazaki-u.ac.jp

**S1: Predation pressure on egg masses**

**Methods S1**

To evaluate the predation pressure of polyphagous snail species, *Tenguella musiva* and *Drupella margariticola*, on the egg masses of the limpet *Siphonaria sirius*, we assessed the predators’ activity seven times during low tides in the daytime (4-Sep-2014; 4, 5, 18, and 19-Aug-2015; 2 and 3-Sep-2015) and three times at nights (3, 4, and 5-Sep-2015) in the intertidal area near the Seto Marine Biological Laboratory, Shirahama, Wakayama, Japan (33°41 N, 135°20 E; Wada et al. 2013, 2015, 2017; Wada and Yusa 2021). At the same time, we also confirmed that the predator of adult *S. sirius*, *Reishia clavigera*, did not eat the egg masses. We evaluated the activity of the predators by counting the total number of individual predators and the number actively feeding on the egg masses. The egg masses of *S. sirius* are shaped like spiral tubes or cylinders (Electronic Supplemental Material, ESM Fig. S1(c)). We removed the predators from the egg masses and checked for the feeding traces left on the spiral-shaped egg mass. In subsequent experiments, we used *T. musiva* (total *n* = 81 in the above survey) as the primary *S. sirius* egg masses predator, as this species was more abundant in the field than *D. margariticola* (*n* = 47). We estimated the feeding rate on 20 egg masses in the field over a 12-hour period on 3 and 4-Sep-2015. Each egg mass was marked by scraping the surrounding rock, photographed with a scale using a camera (PENTAX W80, RICOH, Japan), and later measured the length and width of the egg string using ImageJ (Rasband 2011). One individual *T. musiva* of known shell length was placed on each egg mass. After 12 hours, the presence of predators was checked, feeding traces on the egg masses were examined, and the egg masses were photographed. The volume of each egg mass was calculated using its length and width (diameter), assuming a cylindrical shape, with the following equation:

$$\pi\times length of egg mass\times\left( width of egg mass \right)^{2}\times1/4$$

Then, amount of egg mass consumed was calculated by subtracting the final volume from the initial volume.

**Data and statistical analyses S1**

We performed all statistical analyses using R software (version 4.1.1; R Development Core Team 2021). We used generalized linear models (GLMs) with binomial distributions (logit-link function) to determine whether the presence or absence of egg mass predation depended on the ln(original egg mass volume) and ln(egg predator size). Furthermore, a GLM with a gamma distribution (ln-link function) was used to determine whether the amount of egg mass predation depended on the ln(original egg mass volume) and ln(egg predator size). The analysis of egg mass predation amount was conducted using data from egg masses confirmed to have been preyed upon by predators.

**Results S1**

*T. musiva* and *R. clavigera* were observed during all seven daytime and three nighttime low tides, while *D. margariticola* was found during five daytime and two nighttime low tides. Mean [± SD] of 46.47 [12.08, *n* = 69] % and 44.45 [38.49, *n* = 12] % of *T. musiva* fed on the limpet egg masses during daytime and nighttime, respectively. In contrast, 35.11 [10.50, *n* = 35] % and 81.82 [25.71, *n* = 12] % of *D. margariticola* fed on the egg masses during daytime and nighttime, respectively. In contrast, no *R. clavigera* fed on the limpet egg masses (*n* = 86 for daytime and *n* = 36 for nighttime).

In the feeding experiment, 55 % (11/20 individuals) of *T. musiva* fed on egg masses. The occurrence of feeding did not depend on either the volume of the original egg mass or the size (shell length) of the egg predator (*X*^2^_1_ < 2.47, *P* > 0.12). *T. musiva* individuals that fed on the egg masses consumed 71.47 [22.40] % of the original egg masses within approximately 12 hours. The amount of egg mass consumed did not depend on the predator's size. However, it was significantly influenced by egg mass volumes, and it proportionally increased with increasing egg mass volume (Fig. S2; *b* ± SE = 1.04 ± 0.13, test of *b* = 1, *t_8_* = 0.28, *P* > 0.1).

**Discussion S1**

Our field observations revealed that 46% and 44% of all observed *T. musiva* fed on the egg masses of *S. sirius* during daytime and nighttime low tides, respectively. Similarly, *D. margariticola* fed on 35% and 82% of the egg masses during daytime and nighttime low tides, respectively. In 2014 and 2015, respectively, 44% and 48% of the total egg masses laid on Natural plots, where rocks were maintained in the same condition as their natural state, were preyed upon before hatching. Thus, approximately half of the egg predators in the field preyed upon *S. sirius* egg masses, and approximately half of the egg masses were consumed by these egg predators during the embryonic period. Moreover, a large proportion (70%) of the egg mass was consumed within half a day after being attacked by *T. musiva*. Abe (1983) reported that 51% of the egg capsules (n = 401) of *R. clavigera*, each containing approximately 80 eggs (Lee 1999), were consumed by *Ergalatax contractus* and *D. margariticola* within three days after being attacked. These results indicate that predation on various types of the egg mass of marine gastropods is a common occurrence (Fukumori et al. 2013). At our study site, both *T. musiva* and *D. margariticola* were the dominant predators of *S. sirius* egg masses, potentially exerting strong selective force on the hatching plasticity of *S. sirius*.

**References S1**

Abe N (1983) Breeding of *Thais Clavigera* (Küster, 1858) and predation of its Eggs by *Cronia Margariticola* (Broderip). In: Brian Morton, David Dudgeon (eds) Proceeding of the second international workshop on the Malacofauna of Hong Kong and Southern China. Hong Kong University Press, Hong Kong, pp 381-392

Lee JH (1999) Gametogenesis and reproductive cycle of the rock shell, *Reishia* (*Thais*) *clavigera* (Neogastropoda: Muricidae), on the west coast of Korea. Korean J Biol Sci 3:375-383 doi: 10.1080/12265071.1999.9647511

Fukumori H, Chee SY, Kano Y (2013) Drilling predation on neritid egg capsules by the muricid snail *Reishia clavigera*. J Molluscan Stud 79:139-146 doi: 10.1093/mollus/eyt007

**S2: Long-term effects of adult predators on the prey hatching timing**

**Methods S2**

To confirm the long-term effect of the predator of the adult *S. sirius* on the prey hatching timing, we analyzed unpublished data from Wada et al. (2017). We recorded the hatching timing of *S. sirius* in the presence of the predator *R. clavigera,* which preys on adult *S. sirius,* from 8-Jul to 20-Aug-2013. The adult prey laid egg masses on 13-Aug-2013, and we observed whether the embryos hatched from the egg masses at every daytime low tide from 14 to 20-Aug-2013. Once we confirmed under a microscope that no embryos were present and only egg capsules remained in the egg mass, we concluded that the embryos had already hatched.

To compare differences in hatching timing between the presence and absence of the adult predator cue, we analyzed the results from the predator cue treatments (i.e., plots with both the adult predator and adult prey in a cage; *n* = 9) and the control (i.e., plots with an empty cage; *n* = 9). A generalized linear model (GLM) with Poisson distribution (ln-link function) was used to analyze differences in *S. sirius* hatching timing between the predator cue treatment and control.

**Results S2**

The hatching timing of *S. sirius* did not differ between the presence and absence of the adult predator cue (mean [LSE, USE] = 6.15 [5.91, 6.40], χ^2^_1_ = 0.23, *p* = 0.64).

**S3: Long- and short-term effects of egg predators on the prey hatching timing**

**Methods S3**

To evaluate how the duration of adult *S. sirius* exposure to the egg predators affects their hatching timing, we conducted long-term (29-Jul–31-Aug-2014) and short-term (27–31-Aug-2014) experiments under the egg predation cue in the intertidal area near the Seto Marine Biological Laboratory. We used each rock as a treatment plot, with two treatments (long-term and short-term) conducted across six plots (*n* = 3 per treatment).

In the long-term treatment, a fixed number of egg predators (*T. musiva*) and a fixed proportion of egg masses of *S. sirius* were placed in cages attached to each plot. This treatment was conducted over three periods, starting one day prior to *S. sirius* mating until hatching: the first period (29-Jul to 6-Aug-2014), the second period (13 to 20-Aug-2014), and the third period (27-Aug to 3-Sep-2014). The number of egg predators (*n* = 5, 6, and 6 in the first, second, and third periods, respectively) was constant for each cage. The number of egg predators was determined based on the mean number of predators observed in six rocks without treatment (i.e., Natural plots) just before this experiment (first: 15 to 21-Jul-2014, second: 29-Jul to 6-Aug-2014, third: 13 to 20-Aug-2014). The number of experimental victims (egg masses) placed in each cage was calculated by multiplying the total number of egg masses laid in each plot during the previous egg-laying–hatching period (first: 15 to 21-Jul-2014, second: 29-Jul to 6-Aug-2014, third: 13 to 20-Aug-2014) by the consumption rate of the egg masses (42.13%, 46.75%, and 45.83% of the total egg masses in each plot for the first, second, and third period, respectively). The consumption rates of the egg masses were calculated by dividing the number of egg masses preyed upon by the egg predators (i.e., the egg masses with the egg predators observed on them or visibly consumed) by the total number of egg masses laid in Natural plots during the previous egg-laying–hatching period (first: 15 to 21-Jul-2014, second: 29-Jul to 6-Aug-2014, third: 13 to 20-Aug-2014).

In the short-term treatment, six egg predators and 45.83% of the total egg masses were placed in cages attached to each plot from 27-Aug to 3-Sep-2014 (i.e., the third period of the long-term treatment). The number of egg predators and the consumption rates of egg masses were calculated using data from 13 to 20-Aug-2014, following the same method as in the long-term treatment. The adult limpets mated on 28-Aug-2014 and laid the egg masses on 29-Aug-2014. We observed the embryo hatching during every daytime low tide from 29-Aug to 3-Sep-2014.

A GLM with Poisson distribution (ln-link function) was used to assess the differences in *S. sirius* hatching timing between treatments (long-term vs. short-term).

**Results S3**

No significant difference in hatching timing was observed between long and short-term exposure to the egg predator (mean [LSE, USE] = 5.50 [4.62, 6.54], χ^2^_1_ = 0.03, *P* = 0.86).

**S4: Additive vs. halved treatments**

**Methods S4**

The hatching timing of the prey limpet *S. sirius* was observed under two treatments from 27-Aug to 2-Sep-2014: (1) the additive treatment (*n* = 4), in which the same predator number and prey consumption rate as in each adult predator and egg predator treatment were applied, and (2) the halved treatment (*n* =4), in which both the predator number and the prey consumption rate were halved to ensure that the total predator number and total prey consumption rate were equal to those in each single predator treatment. Six adult and six egg predators were placed in cages for the additive treatment, while three adult and three egg predators were placed in cages for the halved treatment. The predator numbers were based on the mean number of predators observed in Natural plots before this experiment (14 to 22-Aug-2014). The adult *S.sirius* consumption rate (15.52% of total adult individuals in each plot) was estimated by subtracting the average mortality of adult *S.sirius* in Control plots (without predators) from those in Natural plots (with predators) during the previous mating–hatching period of the treatment experiments (13 to 20-Aug-2014). The egg mass consumption rate (45.83% of total egg masses in each plot) was calculated by dividing the number of egg masses preyed on by egg predators (i.e., the egg masses either consumed or with the predators present) by the total number of egg masses laid on Natural plots during the previous egg-laying–hatching period (14 to 21-Aug-2014). The adult limpets and egg masses corresponding to the consumption rates (15.52% and 45.83%, respectively) were placed in the cages for the additive treatment, and half of the adult *S. sirius* (7.76%) and egg masses (22.92%) were placed in the cages for the halved treatment. The adult *S. sirius* laid the egg masses on 29-Aug-2014, and we observed whether the embryos hatched at every daytime low tide from 30-Aug to 2-Sep-2014. A generalized linear model (GLM) was used to determine the differences in *S. sirius* hatching timing between the treatments (additive vs. halved) using Poisson distributions (ln-link function).

**Results S4**

There was no significant difference in the hatching timing of *S. sirius* between the additive and halved treatments (mean [LSE, USE] = 3.63 [3.01, 4.36], χ^2^_1_ = 0.03, *P* = 0.85).

**S5: Number and size of egg**

**Methods S5**

The total number and size of eggs in an egg mass were estimated by cutting three fragments from each egg mass (egg string) after measuring its total weight (*n* = 18 and *n* = 24 fragments data per treatment group in Experiment 1 and 2, respectively). The mean [±SD] weight of the fragments was 0.59 [0.15] mg in Experiment 1 and 1.39 [0.94] mg in Experiment 2. The number of eggs in each fragment was counted, and the length and width of 30 randomly selected eggs per fragment were measured using ImageJ (Rasband 2011). This resulted in *n* = 180 egg data per treatment group in Experiment 1 and *n* = 240 egg data per treatment group in Experiment 2. The total number of eggs in each original egg mass was estimated by extrapolating from the proportion of each fragment’s weight relative to the total egg mass weight (Creese 1980; Ocaña and Emson 1999; Slama et al. 2018). Finally, we calculated the volumes of the egg using the formula for ellipsoid volume, as given below:

$$4/3 \pi\times radius of vertical axis \times(radius of horizontal axis)^{2}$$

**References S5**

Creese RG (1980) Reproductive cycles and fecundities of two species of *Siphonaria* (Mollusca: Pulmonata) in south-eastern Australia. Aust J Mar Freshw Res 31:37-47 doi: 10.1071/MF9800037

Ocaña TMJ, Emson RH (1999) Maturation, spawning and development in *Siphonaria pectinata* Linnaeus (Gastropoda: Pulmonata) at Gibraltar. J Molluscan Stud 65:185-193 doi: 10.1093/mollus/65.2.185

Rasband W (2011). ImageJ, U.S. National Institutes of Health, Bethesda, Maryland, USA.

　　 http://imagej.nih.gov/

Slama T, Lahbib Y, Vasconcelos P, Trigui El Menif N (2018) The alien false limpet (*Siphonaria pectinata*) in the Bizerte channel (northern Tunisia): spawning, development and growth under laboratory conditions. Invertebr Reprod Dev 62:109-118 doi: 10.1080/07924259.2018.1445041


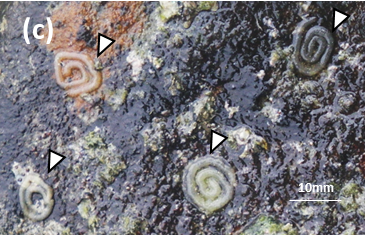

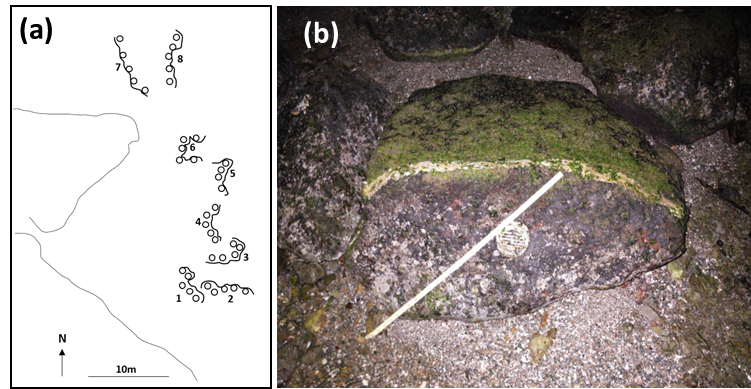


**Fig. S1 (a)** Map of the study site in Shirahama, Wakayama, Japan. The left gray line represents the coastline, with the rocky shore to the right. The numbered lines on the right indicate the blocks, each containing five experimental plots represented by circles. In Experiment 1, six blocks (1, 2, 3, 4, 5, and 8) were used, while Experiment 2 included all eight blocks (1–8). **(b)** An experimental plot established on one side of a rock on sand. The white line surrounding the experimental plot is a copper-infused paint barrier designed to prevent the re-entry of mobile animals that were removed from the plots, except for *Siphonaria sirius*. The cage at the center contains adult predators and experimental victims (adult individuals of *S. sirius* and/or egg masses of *S. sirius*). The response of adult individuals of *S. sirius* and/or embryos of *S. sirius* outside the cage was then evaluated. Ruler = 1 m. (c) Egg masses of *S. sirius* (arrowhead)

**
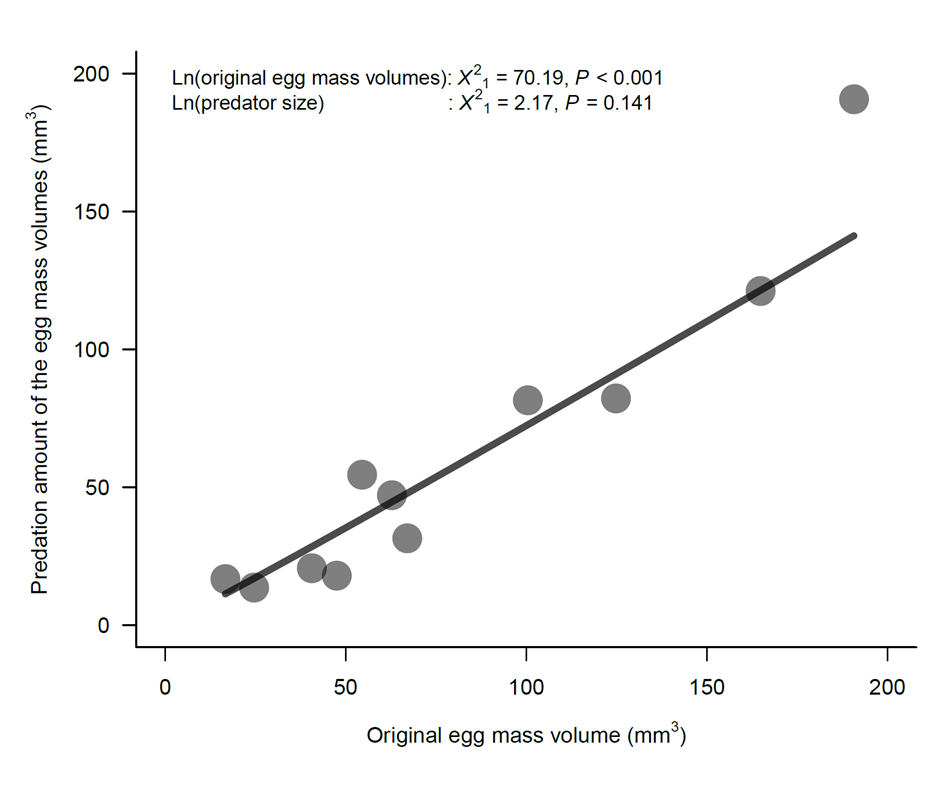
Fig. S2** The effect of the original egg mass volume of *Siphonaria sirius* on the amount of volume eaten by egg predator *Tenguella musiva* (*n* = 11)


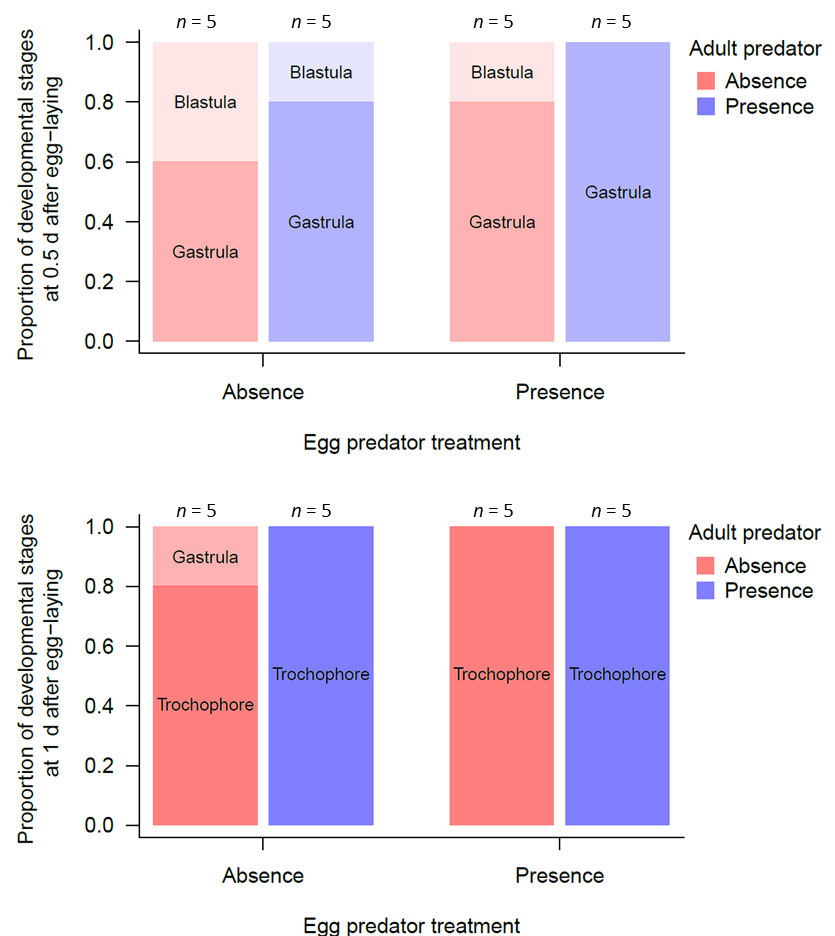


**Fig. S3** The developmental state of *Siphonaria sirius* egg masses in the presence or absence of adult predator *Reishia clavigera* and egg predator *Tenguella musiva* at **(a)** 0.5 days after and **(b)** 1 day after the egg-laying. The developmental state of each egg mass was defined based on the stage attained by the fastest-developing embryos it contains


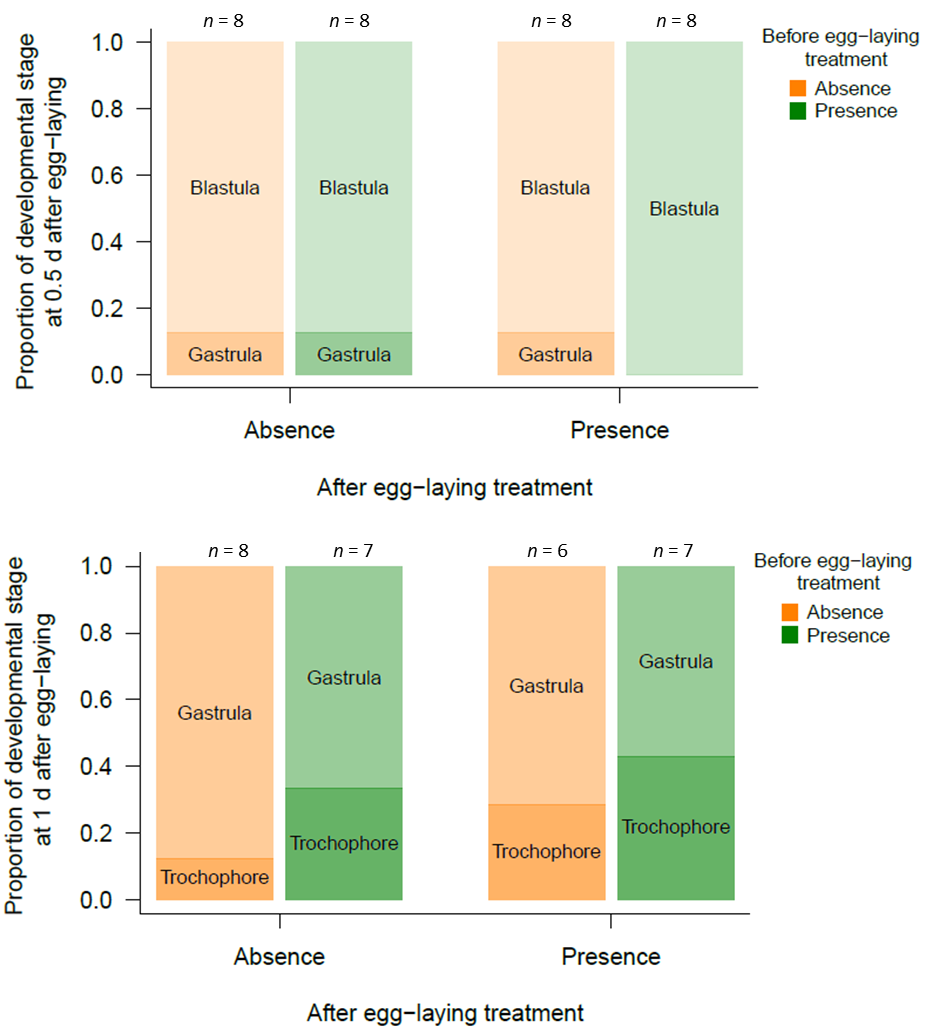


**Fig. S4** The developmental state of *Siphonaria sirius* egg masses in the presence or absence of egg predator *Tenguella musiva* before and after egg-laying at **(a)** 0.5 days after and **(b)** 1 day after the egg-laying. The developmental state of each egg mass was defined based on the stage attained by the fastest-developing embryos it contains
